# Identification and characterization of metabolite quantitative trait loci in tomato leaves and comparison with those reported for fruits and seeds

**DOI:** 10.1007/s11306-019-1503-8

**Published:** 2019-03-15

**Authors:** Adriano Nunes-Nesi, Saleh Alseekh, Franklin Magnum de Oliveira Silva, Nooshin Omranian, Gabriel Lichtenstein, Mohammad Mirnezhad, Roman R. Romero González, Julia Sabio y Garcia, Mariana Conte, Kirsten A. Leiss, Peter G. L. Klinkhamer, Zoran Nikoloski, Fernando Carrari, Alisdair R. Fernie

**Affiliations:** 1 Departamento de Biologia Vegetal, Universidade Federal de Viçosa, Viçosa, Minas Gerais 36570-900 Brazil; 2 Max-Planck-Institute of Molecular Plant Physiology, Am Mühlenberg 1, 14476 Potsdam, Golm, OT Germany; 3 Center of Plant System Biology and Biotechnology (CPSBB), Plovdiv, Bulgaria; 4 Instituto de Biotecnología, Instituto Nacional de Tecnología Agropecuaría, Consejo Nacional de Investigaciones Científicas y Técnicas, B1712WAA Castelar, Argentina; 5 Plant Ecology, Institute of Biology, Leiden University, Sylviusweg 72, 2333 BE Leiden, The Netherlands; 6 Bioinformatics Group, Institute of Biochemistry and Biology, University of Potsdam, Potsdam, Germany; 7 Instituto de Fisiología, Biología Molecular y Neurociencias (IFIBYNE-UBA-CONICET), Universidad de Buenos Aires, Ciudad Universitaria, C1428EHA Buenos Aires, Argentina; 8 Facultad de Agronomía, Cátedra de Genética, Universidad de Buenos Aires, Buenos Aires, Argentina; 9 Present Address: Business Unit Horticulture, Wageningen University & Research, Postbus 20, 2665 ZG Bleiswijk, The Netherlands

**Keywords:** Metabolite QTL, Tomato, Leaf metabolism, Metabolite network

## Abstract

**Introduction:**

To date, most studies of natural variation and metabolite quantitative trait loci (mQTL) in tomato have focused on fruit metabolism, leaving aside the identification of genomic regions involved in the regulation of leaf metabolism.

**Objective:**

This study was conducted to identify leaf mQTL in tomato and to assess the association of leaf metabolites and physiological traits with the metabolite levels from other tissues.

**Methods:**

The analysis of components of leaf metabolism was performed by phenotypying 76 tomato ILs with chromosome segments of the wild species *Solanum pennellii* in the genetic background of a cultivated tomato (*S. lycopersicum*) variety M82. The plants were cultivated in two different environments in independent years and samples were harvested from mature leaves of non-flowering plants at the middle of the light period. The non-targeted metabolite profiling was obtained by gas chromatography time-of-flight mass spectrometry (GC-TOF-MS). With the data set obtained in this study and already published metabolomics data from seed and fruit, we performed QTL mapping, heritability and correlation analyses.

**Results:**

Changes in metabolite contents were evident in the ILs that are potentially important with respect to stress responses and plant physiology. By analyzing the obtained data, we identified 42 positive and 76 negative mQTL involved in carbon and nitrogen metabolism.

**Conclusions:**

Overall, these findings allowed the identification of *S. lycopersicum* genome regions involved in the regulation of leaf primary carbon and nitrogen metabolism, as well as the association of leaf metabolites with metabolites from seeds and fruits.

**Electronic supplementary material:**

The online version of this article (10.1007/s11306-019-1503-8) contains supplementary material, which is available to authorized users.

## Introduction

Integrated approaches combining genomics, transcriptomics, proteomics, metabolomics and bioinformatics have been pivotal for metabolic network reconstruction and complex trait mapping (Saito and Matsuda 2010; Tohge et al. 2015). Moreover, several studies suggest that metabolite levels are heritable and might be controlled by multiple genes (Schauer et al. 2008; Chan et al. 2010; Alseekh et al. 2015; Wen et al. 2015) and can therefore be considered as quantitative traits regulated by metabolite quantitative trait loci (mQTL). Some groups have reported genome wide association studies in tomato fruits (Sauvage et al. 2014; Tieman et al. 2017) or on leaf metabolism for a range of wild species (Schauer et al. 2005b; Matsuba et al. 2013; Ning et al. 2015; López et al. 2015). To date, most mQTL research has been carried out by investigating introgression lines (ILs) or recombinant inbred lines (Schauer et al. 2006, 2008; Zanor et al. 2009; Do et al. 2010).

In tomato, researchers have extensively used a population of ILs of the wild species *S. pennellii* in the genetic background of the cultivated variety M82 of *S. lycopersicum* (Eshed and Zamir 1995). In this population, marker-defined genomic regions of *S. lycopersicum* were replaced by single homologous intervals of the *S. pennellii* comprising a core set of 76 lines. This population has been extensively phenotyped and allowed the identification of multiple QTL related to different traits. For example, some of the identified QTL were linked to fruit primary metabolite composition (Causse et al. 2004; Baxter et al. 2005; Schauer et al. 2006), fruit color (Liu et al. 2003) and enzymatic activity (Steinhauser et al. 2011). Others were associated with volatile metabolites and antioxidants (Baxter et al. 2005; Tieman et al. 2006; Quadrana et al. 2014), organ morphology (Holtan and Hake 2003; Chitwood et al. 2013a; Ron et al. 2013), tolerance to abiotic and biotic stress (Lippman et al. 2007). Other QTL of ILs were associated to leaf water-use efficiency (Xu et al. 2008) stomatal responsiveness, and stomatal-related anatomical traits (Fanourakis et al. 2015), seed composition (Toubiana et al. 2012, 2015) and secondary metabolite abundance (Alseekh et al. 2015; Schilmiller et al. 2015).

Recently we used the *S. pennellii* ILs to characterize photosynthetic and respiratory parameters, including CO_2_ assimilation rate, stomatal conductance, chlorophyll *a* fluorescence parameters, growth traits and the main leaf primary metabolites (de Oliveira Silva et al. 2018). The findings of that study revealed a wide natural variation in plant growth, photosynthetic and essential metabolites (related to carbon and nitrogen metabolism) among tomato ILs. In total, over 3069 QTL have been identified in this population to date (reviewed in Alseekh et al. 2013) and a handful of these QTL have been cloned (Pnueli et al. 1998; Ronen et al. 2000; Fridman et al. 2004; Quadrana et al. 2014). The recent genome sequencing of the parental lines of this population (Bolger et al. 2014) should facilitate this labour-intensive process.

The combination of mQTL analysis with genomic technologies (Ingvarsson and Street 2011) allows researchers to provide lists of candidate regulatory genes of the metabolic variations as well as of their effects on physiological parameters and interactions (Keurentjes et al. 2006). This approach, therefore, could widen the understanding of the genetic regulatory networks that might influence plant performance and yield (Jansen et al. 2009; Hall et al. 2010).

In the present study, we analyzed the metabolite contents of mature leaves from non-flowering tomato plants harvested at the middle of light period. The analysis was performed in two consecutive harvests of the *S. pennellii* ILs. We contrast the results herein obtained to those previously reported for fruit and seed metabolites in the same population and with our recent analysis on the physiological properties of photosynthesis and respiration, also within this same population.

## Materials and methods

### Plant materials and experimental conditions

Seeds from 76 *S. pennellii* ILs (Eshed and Zamir 1995; Liu and Zamir 1999) and *S. lycopersicum* M82, kindly provided by Professor Dr. Dani Zamir (Hebrew University, Rehovot, Israel), were used in this study. In this IL population, unique overlapping genomic regions between introgressions delimitate shorter intervals than the ones defined by the ILs, named “Bins” (Chitwood et al. 2013a, b). In total, 112 Bins have been defined by the complete set of ILs and most of the Bins harbor fewer than 500 annotated genes, with an average of 295 genes in each region (Chitwood et al. 2013a, b).

The seeds were germinated on MS medium (Murashige and Skoog 1962) containing 2% (w/v) sucrose and were grown in a growth chamber (250 µmol photons m^−2^ s^−1^, 22 °C) under a photoperiod of 16 h-light/8 h dark before transfer into the greenhouse. Once rooted, plantlets were acclimatized and subsequently transferred to 1.16 L pots with potting soil as previously described (Mirnezhad et al. 2010). High variability for metabolite data is generally observed because of changes in environmental conditions (Schauer et al. 2006, 2008; Harrigan et al. 2007; de Oliveira Silva et al. 2018). For this reason, to access the contribution of environment, we repeated the experiment and analysis in two independent experiments, in two locations and selected the ILs with conserved changes (de Oliveira Silva et al. 2017). In the first year of experiment, four plants for each line were grown in a randomized fashion in a glass house at the Plant Ecology Department, Institute of Biology, Leiden University, Leiden, Netherlands. In the second year, four replicates for each line were also grown in a randomized fashion in a glass house at the Instituto de Biotecnologia, Instituto Nacional de Tecnologıa Agropecuaria (IB-INTA), in Castelar, Argentina. In both experiments, the plants were grown in the spring season under greenhouse conditions with a 16 h/8 h photoperiod, at 24 ± 5 °C, with 60% humidity and 140 ± 40 µmol photons m^−2^ s^−1^ incident photo-irradiance. Tomato source leaves were sampled from the second or third leaflets of the third fully expanded leaf of 4 week-old non-flowering plants.

### Determination of metabolite levels

Leaf samples were harvested at the middle of the light period, immediately frozen in liquid nitrogen and stored at − 80 °C until further analysis, as recommended by Fernie et al. (2011). Relative metabolite contents were determined using an established gas chromatography time-of-flight mass spectrometry (GC-TOF-MS)-based protocol. This protocol allows the quantification of sugars, sugar alcohols, organic and amino acids from the methanol extracts (Roessner-Tunali et al. 2003; Lisec et al. 2006). Chromatograms and mass spectra were evaluated by Chroma TOF® 4.2 (Leco, St Joseph, MI) and TagFinder 4.0 (Luedemann et al. 2012). Analytes were manually identified using TagFinder by comparing the results with the reference library mass spectra and retention indices in the Golm Metabolome Database (Kopka et al. 2005). These procedures have been previously optimized for tomato (Roessner-Tunali et al. 2003). Mass-spectral libraries (Schauer et al. 2005a; Kopka et al. 2005) were used for peak identification. The use of these libraries allowed us to detect up to 78 metabolites (Supplementary Table 1), a slightly larger number of metabolites than in our previous study (Schauer and Fernie 2006).

### Heat maps

Heat maps were generated by using the heatmap function of the statistical software environment R, version 1.9. False color imaging was performed on the log2-transformed metabolite data using x-fold changes to cultivated variety M82.

### QTL mapping and heritability

This procedure was carried out as previously described (Schauer et al. 2008). The broad-sense heritability (H^2^) was estimated by mixed effect models, with random effects for genotype (number of ILs), environment (years 2001 and 2004) and genotype-environment interaction. We used the lmer function from the lme4 package in the R environment. The genetic variation coefficient $Cvg(\% )=(100{\sqrt {{\sigma ^2}} _g})/mc$ and coefficient of environment variation $- \,Cve(\% )=(100{\sqrt {{\sigma ^2}} _e})/mc$ where, $\sigma _{g}^{2}=\frac{{QMg - QMe}}{r}$ and $\sigma _{e}^{2}=\frac{{QMe}}{r}$ *r* and *mc* correspond to repetitions number and average of each metabolite, respectively were obtained for each of the analyzed metabolite with the Genes program (Cruz 2013).

For QTL mapping, each IL was compared by *t*-test with M82 for each harvest. If an IL was significantly different from the reference genotype M82 (5% level) in both experiments and in the same direction, the effect was considered as harboring a QTL.

### Network analysis

#### Pre-processing metabolite data

The metabolite data sets were preprocessed following different steps. First, (i) the lines have been filtered out for which none of the metabolites were detectable. Also (ii) if the concentration of a specific metabolite was unavailable for more than half of the lines, the metabolite was filtered out. Finally, (iii) the rest of the missing values were imputed using random forest imputation (from randomForest package in R (Liaw and Wiener 2002)). To be robust with the estimation of missing values, we repeated the imputation 10 times and used the average values to impute the NA values.

#### Metabolite relevance network

The metabolite relevance networks were separately inferred for each tissue (leaf, seed and fruit) in each and between the two seasons. To infer the networks, we applied pairwise Pearson correlation with the coefficients at a significance level of 0.01 (FDR corrected) to appear as a link in the network. The list of links for each network is presented in Supplementary Table 2.

#### Morphological trait-metabolite association network

The same preprocessing procedure as for metabolites data was applied on phenotypic trait data. The missing values were imputed similarly. The relationships between all measured metabolites in leaf and the phenotypic traits were obtained by employing elastic net regression (cv.enet from elasticnet package in R (Zou and Hastie 2012). The profiles of the same metabolites from leaf in both seasons were concatenated and considered as predictors (regressors). Regression models were fitted for each phenotypic trait by combining the corresponding profiles from both seasons. The regression coefficients were robustly estimated by 10-fold cross validation based on the optimum value for the penalty parameter from the set (0.01, 0.05, 0.1, 0.5, 1, 1.5, 2, 10, 100). The r-squared, which measures how well the data fitted to the regression model, was calculated for each model and represented in the network with the size of the corresponding node (the bigger the size of the node, the better fitted the model to the data). The list of the links in the morphological trait-metabolite association network is represented in Supplementary Table 3.

## Results

### Evaluation of leaf mQTL in the *S. pennellii* ILs

To assess the natural variation in leaf primary metabolism, we employed an established GC-TOF-MS based metabolite profiling platform (Lisec et al. 2006) to the *S. pennellii* ILs (Eshed and Zamir 1995). Although this strategy has already been applied to field grown fruit material (Schauer et al. 2006) and seeds derived thereof (Toubiana et al. 2012), it has not yet been applied to leaf material. This was in part because leaves of field grown plants were considered too heterogeneous to provide reliable material. This is the reason why we decided to evaluate greenhouse grown material here.

In total, we identified 45 and 78 metabolites in Experiments 1 and 2, respectively. The complete datasets are provided in Supplementary Tables 4 and 5. The two data sets evidenced a large variability between the 2 experiments for all analysed metabolite classes (Supplementary Table 6). Surprisingly, the total number of significant changes was substantially higher in Experiment 1 than in Experiment 2 (995 and 338 respectively). By counting the number of the ILs that exhibited the same alteration in both experiments, we verified that the highest percentage of ILs with stable changes in amino acids levels occurred for threonine, asparagine and glutamate (10.8, 9.8 and 8.6%, respectively). The highest percentage of ILs with conserved changes for organic acids was for 2-oxoglutarate and succinate (17.0 and 13.5% respectively), whereas for sugars was for glucose and maltose (9.4 and 4.8%, respectively). Regarding the changes in the content of metabolites from other classes, phosphoric acid, 3-caffeoyl-quinic acid (cis), glycerol and trehalose were the compounds with most conserved changes between the two locations (20.7, 15.4, 13.6 and 10.2% respectively). These results indicate a strong environmental component, probably due to substrate composition and other seasonal related effects, for the analysed metabolites in leaves and suggest that most changes in metabolite levels were adaptive and not constitutive.

To test how genetic variation may influence metabolite variation in this IL population, we estimated the genotypic coefficient of variation (*Cvg*) per metabolite (Supplementary table 7). The average *Cvg* of all metabolite traits in both experiments was 77.6 and 73.2% in Experiment 1and 2, respectively. By corollary, the coefficients of environmental variation (*Cve*) for Experiment 1 and 2 were therefore 22.4 and 26.8% for Experiments 1 and 2, respectively. Thus, even though we attempted to keep non-experimental variables constant, environmental differences were apparent. These environmental variables, however, were considerably less variable quantitatively than the genotype-derived variance.

**Fig. 1:**
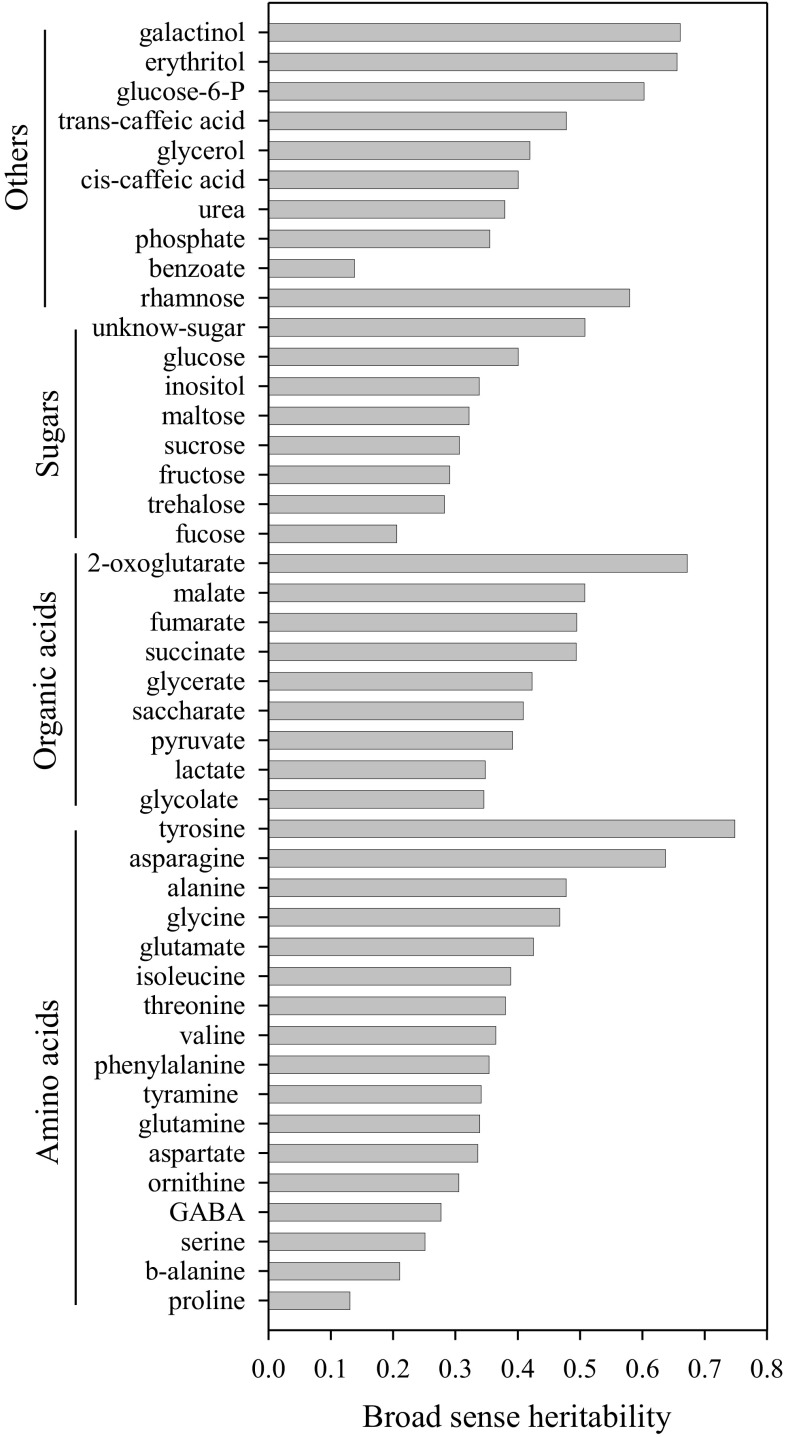
Broad sense heritability of leaf metabolite traits of population of introgression lines of *Solanum pennellii* into a genetic background of *Solanum lycopersicum* (M82)

We next assessed the heritability (H^2^) of each metabolite trait (Fig. 1). The measured traits exhibited mean H^2^ between 0.13 and 0.75 – corresponding to amino acids proline and tyrosine, respectively. Of the measured sugars, an unknown-sugar and rhamnose displayed moderate to high H^2^ values of 0.51 and 0.58, respectively. The metabolites 2-oxoglutarate (0.67) and asparagine (0.64) showed high H^2^ values, whereas malate, fumarate and succinate displayed moderate H^2^ with values of 0.51, 0.49 and 0.49, respectively. Taken together, these analyses suggest that the genetic variation is a major component controlling the metabolites from tomato leaves and that leaf metabolite abundances are moderately heritable in this species.

### Identification of mQTL

**Fig. 2:**
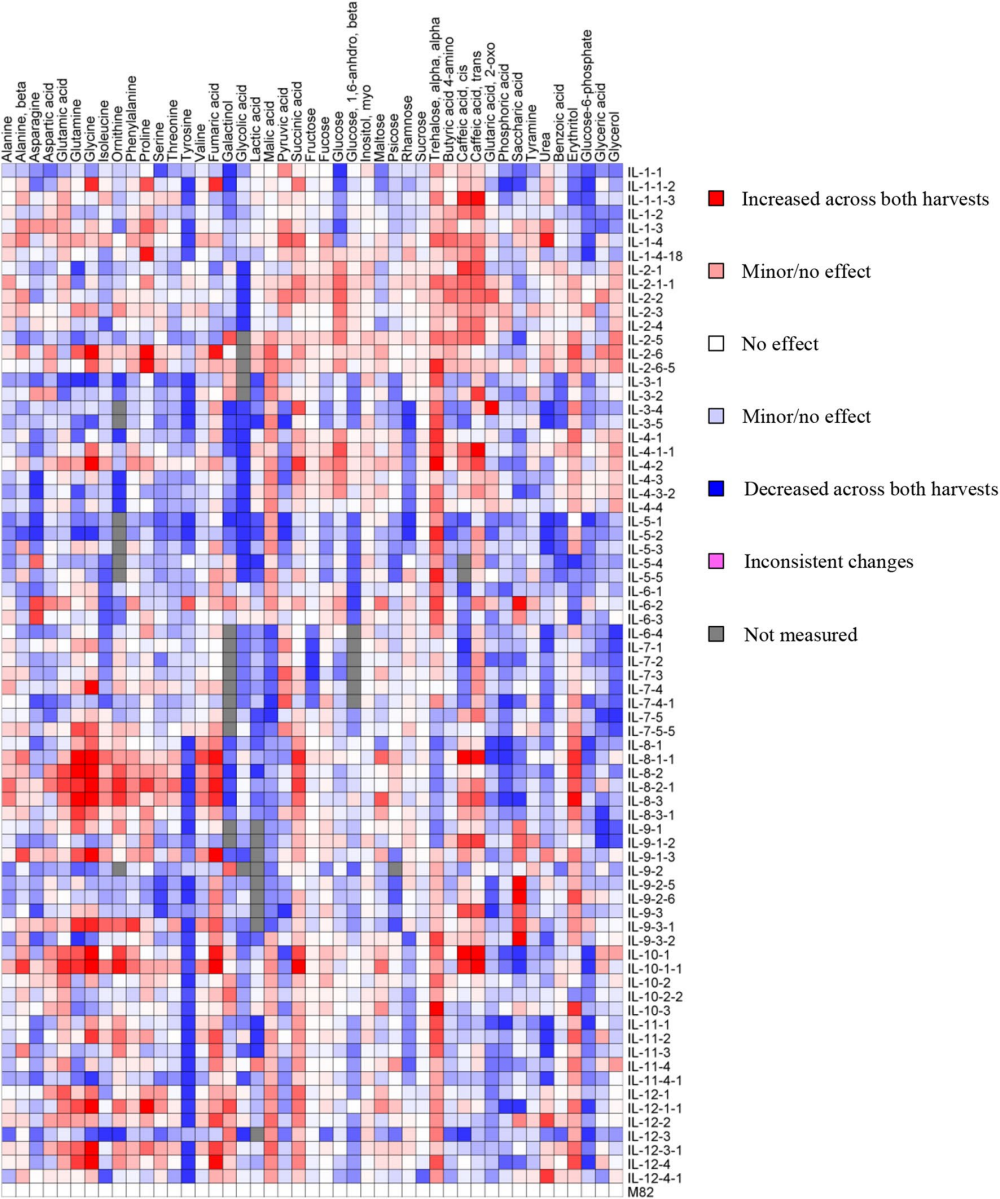
Overlay heat map of the metabolite profiles of two independent studies of the leaf metabolite content of the ILs compared with the parental M82. Data represent measurements of leaf material harvested from two independent experiments presented as a heat map. Large sections of the map are white or pale in color and reflect that many of the chromosomal segment substitutions do not have an effect on the amount of every metabolite. Regions of red or blue indicate increased or decreased metabolite content, respectively, after introgression of *Solanum pennellii* segments. Very dark coloring indicates that a large change in metabolite content was conserved across both harvests, whereas purple indicates an inconsistent change in that IL relative to M82. For each harvest, GC-TOF-MS was used to quantify primary metabolites, including amino acids, organic acids, sugars and other metabolites. The ILs are presented in chromosomal order from top of chromosome 1 to base of chromosome 12 from top to bottom. A total of 76 ILs and 42 metabolites overlapped in both experiments

Using GC-TOF-MS, we were able to quantify 42 and 76 metabolites, including a minimum of 15 amino acids, 10 sugars, seven organic acids and 13 miscellaneous compounds, in the leaves of *S. pennellii* ILs from two successive experiments. The full data sets are presented in an overlay heat map from samples harvested in the Experiment 1 and 2 (Fig. 2). The data sets were superimposed on one another in an additive manner. In this way, consistently large increases, with respect to the *S. lycopersicum* content, are displayed in a deep red color, whereas consistently large decreases are shown in a deep blue color. Finally, those that increase in one year and decrease in the other are displayed in a purple color (for details see Supplemental Figs. 1 and 2 and Supplemental Tables 3 and 4).

The relative differences in the content of any given metabolite range between 0 (below the levels of detection) and a 332-fold increase compared with the recurrent parental cultivar M82. The evaluation of the entire data set indicated that the amino acids glycine and proline exhibited the greatest differences followed by fumarate and *trans*-caffeic acid, whereas threonine, asparagine and saccharic acid exhibited the lowest levels in comparison to the parental cultivar M82.

### Genome location of the identified mQTL

**Fig. 3:**
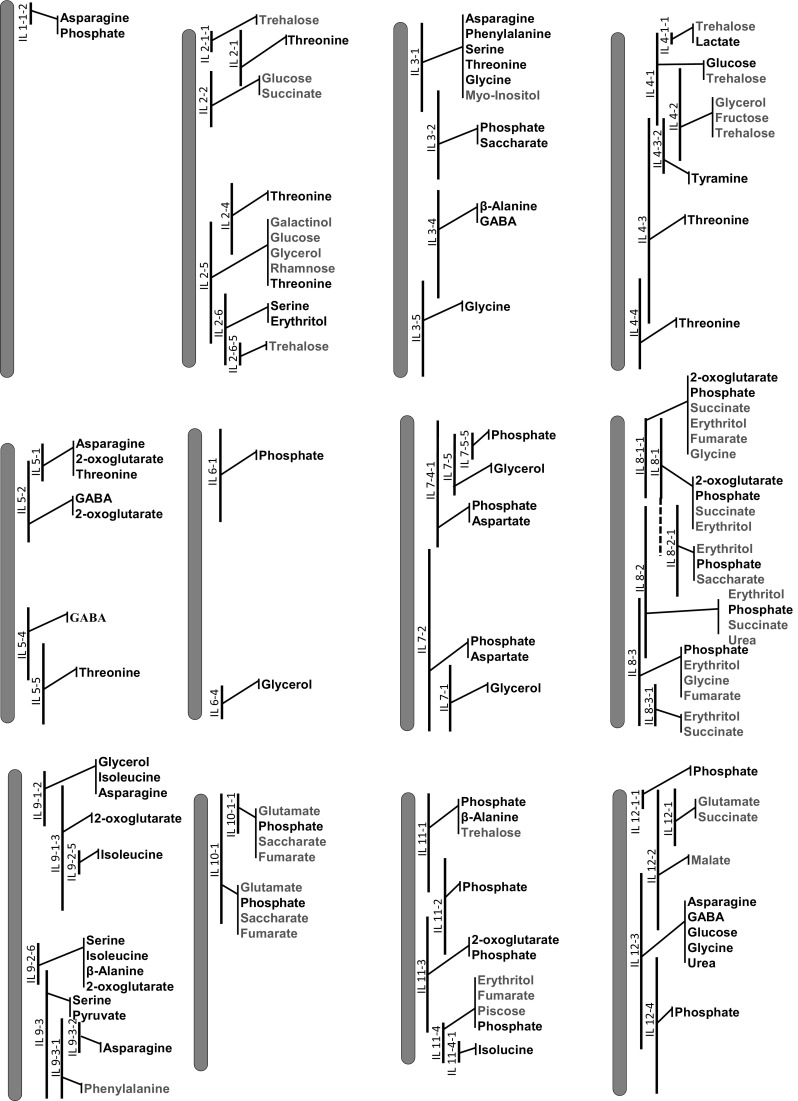
QTL map of significant changes conserved in the metabolite traits in both experiments (Exp1 and 2) of *Solanum pennellii* ILs (black bar) into a genetic background of *Solanum lycopersicum* (M82). Red and blue indicate increased and decreased levels of metabolites, respectively, in relation to M82, as identified by t-test (p value ≤ 0.05)

We next used the data for mapping mQTL using ANOVA tests at a significance threshold of 0.05 to statistically compare each IL with the control M82. Only a subset of the ILs displayed significant differences in metabolite content from the control M82 in both years of experiments. The following analyses were focused on these mQTL. By using this criterion, we identified 118 mQTLs that were conserved in both experiments (Fig. 3; Supplemental Fig. 3). We then calculated the total number of genes within each identified QTL as previously described (Chitwood et al. 2013a, b) (Supplementary table 8) and found that the identified mQTL ranged from 98 to 2185 genes.

The evaluation of these data, regarding compound classes, indicated that 32 mQTL were for amino acids, 23 for sugars 27 for organic acids and 35 for other metabolites. In addition, the evaluation of the distribution of mQTL across the genome (Fig. 3) indicated that the conserved mQTL are unevenly distributed across the tomato genome, with fewer mQTL on chromosome 1 and 6 (2 mQTL for each chromosome). The highest number of mQTL were on chromosome 8 (23 mQTL) as well as on chromosome 2 and 9 (13 mQTL each).

Although most of leaf mQTL reported here were unknown, some mQTL have been identified previously for tomato fruits (Schauer et al. 2006, 2008) or seeds (Toubiana et al. 2012, 2015) (Supplementary Table 9). For example, the IL2-5 and IL4-3 exhibited lower levels of threonine in leaves, fruits and seeds, whereas the IL5-2 and IL12-3 had lowered content of GABA in leaves and exhibited higher contents of the same metabolite in fruits. Interestingly, 19 ILs displayed lower levels of phosphate in leaves, although 5 of these ILs had higher phosphate content in seeds or fruits.

### Morphological trait-metabolite association network

**Fig. 4:**
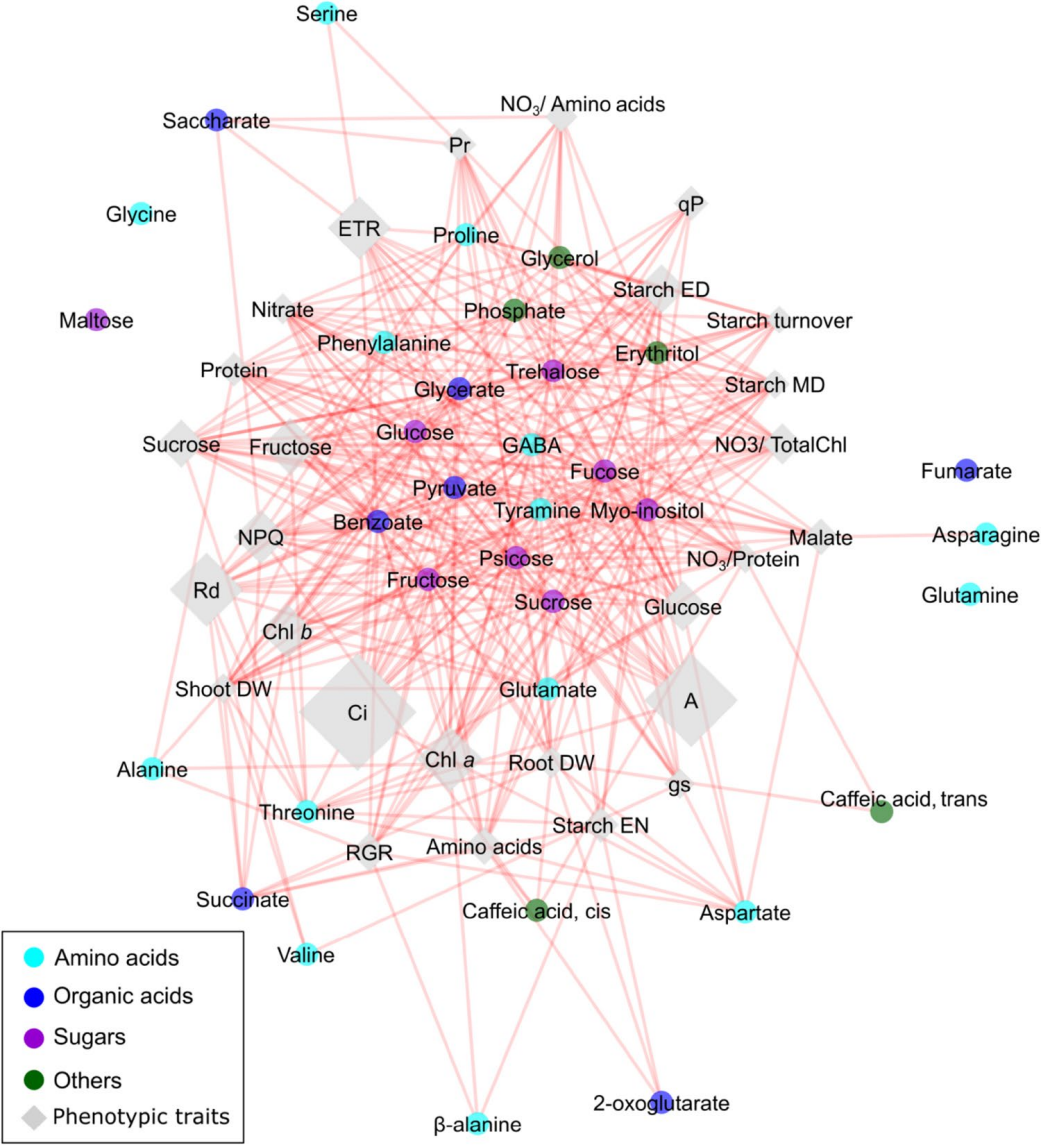
Morphological trait-metabolite association network obtained for leaf. The red and blue links imply positive and negative correlation coefficients, respectively. The color of the nodes represents the metabolite classes. The “diamond” shape in the nodes stands for phenotypic and metabolite traits (from: de Oliveira Silva et al. 2018), whereas the “circle” shape corresponds to the metabolites in leaf. The r-square, which measures how well the data fitted to the regression model, was calculated for each model (phenotypic trait) and represented in the network with the size of the corresponding node. The bigger the size of the diamond, the better the model fitted to the data

Here we used the morphological traits measured on the leaves of the ILs of our recent work (de Oliveira Silva et al. 2018) to conduct leaf morphological trait-metabolite associations. The regression models have been fitted for each phenotypic trait by combining the corresponding profiles from both seasons (see materials and methods for detail). Figure 4 shows the network associations analysis between the 27 physiological/morphological traits (de Oliveira Silva et al. 2018) and metabolite measured by GC-TOF-MS in this study. The links in the morphological trait-metabolite association network are presented in the Supplementary table 3.

The results revealed a strong correlation between intercellular CO_2_ (C_i_), electron transport rate (ETR), chlorophylls and dark respiration (R_d_) and several metabolites (Fig. 4). Among those, the sugars sucrose, psicose-like sugar, fructose and *myo*-inositol are the metabolites most linked to C_i_. A similar trend is evident between the ETR and metabolites glucose, GABA and *myo*-inositol.

**Fig. 5:**
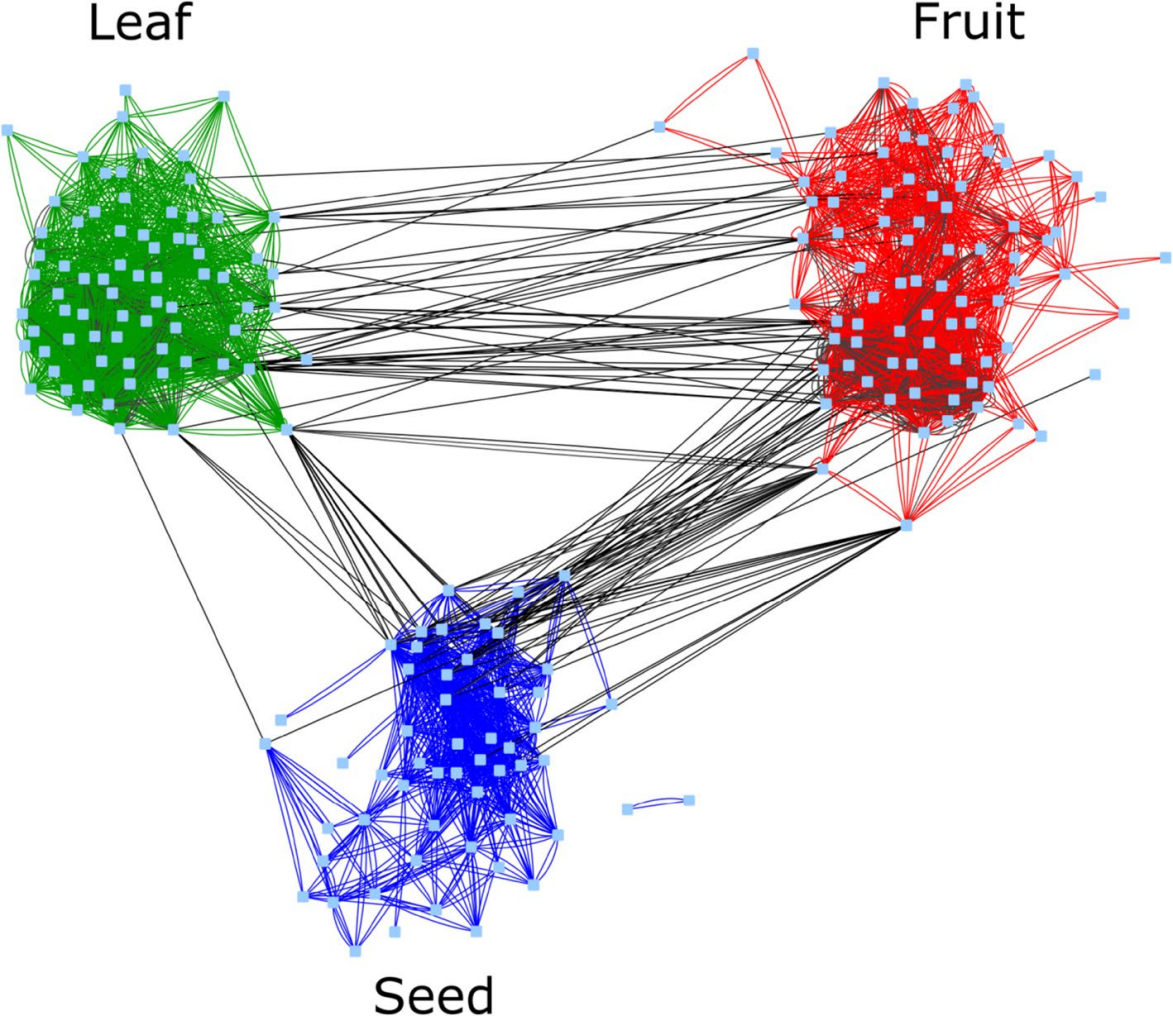
Leaf-fruit-seed metabolite relevance network. Metabolite relevance networks have been inferred for each tissue. Green, red and blue edges correspond to the tissue specific relationships between metabolites in leaf, fruit, and seed, respectively. Black edges indicate the relationship between metabolites from different tissues

We next assessed the correlation between the metabolite levels in leaves (this study), fruit (Schauer et al. 2006) and seed (Toubiana et al. 2012) (Fig. 5; Supplementary Table 2). Interestingly, the most highly correlated parameters are those within the same tissue i.e. leaf–leaf, fruit–fruit and seed–seed parameters. However, there was relatively little correlation between the actual levels of metabolites between tissues. For examples, galactinol and 2-oxoglutarate are the most highly correlated between leaf and fruit (0.82–0.98, *p* < 0.05) such as amino acids (e.g tyrosine, alanine, homoserine, asparagine, isoleucine, valine, serine, methionine, threonine), organic acid; fumarate and glycerate, and others like calysteg A3 and squalene, also the 2-oxoglutarate showed the highest number of correlation between the leaf and seeds data mainly with β-alanine, asparagine, GABA, arginine, lysine and galacturonate.

## Discussion and conclusion

Great effort has gone into characterizing the tomato fruit metabolome, with a focus on spatio-temporal changes in metabolism (Carrari et al. 2006; Mounet et al. 2009; Osorio et al. 2002) as well as on primary and secondary metabolism following both classical quantitative trait loci approaches (Schauer et al. 2006; Tieman et al. 2006; Do et al. 2010; Alseekh et al. 2015; Ballester et al. 2016; Garbowicz et al. 2018). More recently, some groups have reported genome wide association studies (Sauvage et al. 2014; Tieman et al. 2017; Ye et al. 2017) and others have assessed the effects of domestication and crop improvement on the metabolome. For instance, the selection for alleles of genes associated with larger fruits altered metabolite profiles as a consequence of linkage with nearby genes. In addition, the selection of 5 major loci reduced the accumulation of anti-nutritional steroidal glycoalkaloids in ripened fruits, thus rendering the fruit more edible. On the other hand, breeding for pink tomatoes modified the content of over 100 metabolites. That said, with the exception of exquisite work carried out dissecting acyl sugar metabolism in trichromes (specialized secretory cell types found in leaves; see for example (Fan et al. 2017)), relatively little work has been performed on the characterization of leaf metabolism of the *S. lycopersicum* complex (Schauer et al. 2005b; de Oliveira Silva et al. 2018).

With this in mind, we used GC-TOF-MS to evaluate the levels of primary metabolites in the well-studied *S. pennellii* ILs (Eshed and Zamir 1995; Alseekh et al. 2013) grown in two independent greenhouse trials.

Despite the relative high broad sense heritability of most metabolites, with most values being of similar magnitude to those published previously for fruits (Schauer et al. 2006), far fewer QTL were identified for the leaves (117) than for the fruit (889). Intriguingly, this occurs even when the maximal variance in compound abundance is far greater for leaf samples described here than for either the fruit or seeds (Toubiana et al. 2012) of this population. There are several possible explanations for the reduced identification of QTL. Leaf metabolite content may be more influenced by the environment because it is more closely related to photosynthesis than the fruit, which largely relies on leaf assimilate supply, even though it can, at least during early stages of fruit development, produce its own photoassimilate (Lytovchenko et al. 2011). Another possible explanation is that when we analyzed gene expression datasets (Chitwood et al. 2013b), they were far considerably less changed in the leaves – a fact that is true both of all genes and only those genes associated with metabolism (Supplementary Fig. 4). Whatever the explanation, the lower QTL number in leaves indicates that leaf metabolite levels are likely under far greater environmental, and probably also genetic, influence than those of the fruit or seed. The degree of canalization of metabolism may be far lower than that we recently reported in the fruit (Alseekh et al. 2017). Further harvests will need to be evaluated before we can formally test this hypothesis. However, the high influence of environment on the metabolome notwithstanding, a number of interesting novel QTL were detected here. At the genome level, the 118 QTL were largely well-spread across the genome with the individual lines containing between 0 and 6 QTL. However, chromosome 2, 8 and 9 could be regarded as metabolite QTL hotspots similar to those previously reported in the genomes of Arabidopsis, rice and maize (Lisec et al. 2009; Wen et al. 2015; Li et al. 2016). Interestingly, a previous survey of the *S. lycopersicum* complex revealed that the parental lines of the ILs displayed highly divergent primary metabolite abundances. In that survey, the amino acids alanine, β-alanine,  glutamine, glutamate, glycine, proline and serine were considerably higher in *S. pennellii*, whereas GABA and the branched chain amino acids were considerably higher in *S. lycopersicum* (Schauer et al. 2005b). On the other hand, the organic acids ascorbate and citrate were much higher and isocitrate and 2-oxoglutarate much lower in *S. pennellii* than in *S. lycopersicum* and, finally, glucose was much higher but other sugars much lower in *S. pennellii* (Schauer et al. 2005b). Whereas many of the directionalities of the QTL correspond to the differences between the parental lines, this is, by no means, always the case. For example, there are QTL for decreased glucose content and increased minor sugar contents, suggesting the presence of a considerable degree of transgressive behavior, as previously observed in the fruit (Schauer and Fernie 2006). Furthermore, here we identified mQTL that have been identified previously for seeds (Toubiana et al. 2012, 2015) and fruits (Schauer et al. 2006, 2008) (Supplementary Table 8).

We were also interested in how the metabolite QTL were related to the 27 physiological and morphological phenotypes we have previously collected on this population (de Oliveira Silva et al. 2018). Highly interesting correlations were evident and, because of the size of the population, are likely very robust. Amongst these are the strong correlations between the sugars sucrose, fructose and *myo*-inositol with the intracellular carbon dioxide concentration (Ci). In addition, glucose, GABA and *myo*-inositol were strongly correlated with the chloroplastic ETR. Similarly, sucrose, glucose and fructose as well as the amino acids threonine, alanine and aspartate were highly correlated with shoot dry weight. These correlations are all consistent with the role of leaves as a source tissue. Indeed, the links to Ci and the ETR likely reflects that high levels of the latter result in high photoassimilate production but the effects on dry weight to the shoots likely reflecting the fact that sugars are available as substrates for polymer synthesis and respiration, respectively.

Perhaps equally interestingly, some of the correlations that we had anticipated were not detected here. For example considerable evidence links the abundance of both ascorbate and malate to various aspects of photosynthesis with transgenic tomato plants that accumulate different levels of these metabolites and show alterations in CO_2_ assimilation rates and stomatal aperture (Nunes-Nesi et al. 2005; Araujo et al. 2011) and to studies in experimental vineyard; which suggests the generality of this relationship (Gago et al. 2017). These relationships were not uncovered in our current study. This may however merely reflect the relatively minor changes in the leaf levels of malate and ascorbate across the population.

Noteworthy, our study also demonstrated that a greater number of highly correlated parameters occur within the same tissue and not between tissues (Fig. 5). This result is at first glance surprising, particularly because in our previous study the harvest index (the ratio of agronomically useful yield over the total yield) exhibited a controlling role in fruit metabolite composition (Schauer et al. 2006; Phuc et al. 2010). It is important to mention that the plants were not at a similar developmental stage when seed/fruit and leaf tissues were harvested. This certainly added to the low number of correlations observed between metabolites from different organs but highlighted the leave metabolites that are highly associated with fruit and seed metabolites even in early stages of plant development. For example, succinate and 2-oxoglutarate in leaves of young plants are highly associated with several metabolites in seeds (Fig. 5). Moreover, because fruit, seed and leaf exhibit distinct metabolic activity and have different degree of specialization, the observed results highlight the existing differences in metabolite composition between different plant tissues and organs, as previously described (Desbrosses et al. 2005; Matsuda et al. 2009, 2010). These studies emphasize that plants synthesize hundreds to thousands of compounds, of which some are accumulating in specific and distinct tissues and organs, whereas others are more general and concentrate in several tissues. Thus, our results suggest that flexibility in the regulation of metabolism is certainly required for the dynamic control of metabolic activity among the tissues and organs.

In summary, here we have characterized the genetic architecture of primary metabolites within the tomato leaf, extending the data of the far more studied fruit and seed tissues. A whole plant understanding of metabolism will be required to optimize crop yield and quality in next generation crop engineering (Sonnewald and Fernie 2018). In agreement, similarly to other such cross tissue analyses performed in maize (Wen et al. 2015), 10 of these QTL are conserved across the tissues, whereas many others are tissue specific. The availability of the genome sequences of both parental lines (Bolger et al. 2014) together with a higher resolution backcrossed inbred lines population of *S. pennellii* likely represent important tools for the understanding of the individual metabolite QTL and their associations with physiological and morphological traits.

## Electronic supplementary material

Below is the link to the electronic supplementary material.

Supplementary material 1 (3D 507 KB)

Supplementary material 2 (PPTX 711 KB)

Supplementary material 3 (PPTX 296 KB)

Supplementary material 4 (PPTX 90 KB)

Raw metabolite data of leaves of *Solanum pennellii* introgression line (IL) population and parental control (*Solanum lycopersicum* cv. M82). Data represented as fold changes of metabolite content of each individual IL replicate compared with control (cv. M82) of Experiment 1 (Leiden, The Netherlands) and Experiment 2 (Buenos Aires, Argentina)—Supplementary material 5 (XLSX 624 KB)

List of links for each network. The metabolite relevance networks have been separately inferred for each tissue (leaf, seed and fruit) and between the two seasons. The digits 1 and 2 stand for the first and second experiments, respectively. For fruits, the data from the third trial were also available and the digit 3 refers to the metabolite data obtained from the tissue fruit in the third trial. To infer the networks, we applied pairwise Pearson correlation and coefficients at a significance level of 0.01 (FDR corrected) were selected to appear as a link in the network—Supplementary material 6 (XLSX 529 KB)

Links in the morphological trait-metabolite association network. The r-squared, which measures how well the data fitted to the regression model, has been calculated for each model and represented in the network with the size of the corresponding node (the bigger the size of the node, the better fitted the model to the data). For details see Material and Methods—Supplementary material 7 (XLSX 20 KB)

Leaf metabolite QTLs identified for different ILs in Experiment 1. Significant metabolites identified by t-test (p-value≤ 0.05) as applied to IL leaves harvested at the middle of the light period from four-week old plants in Experiment 1 in Leiden, The Netherlands, in comparison with the control M82. Each value represents fold change as compared to M82—Supplementary material 8 (XLSX 127 KB)

Leaf metabolite QTLs identified for different ILs in Experiment 2. Significant metabolites identified by t-test (p-value≤ 0.05) as applied to IL leaves harvested at the middle of the light period from four-week old plants in Experiment 2 in Buenos Aires, Argentina, in comparison with the control M82. Each value represents fold change as compared to M82—Supplementary material 9 (XLSX 208 KB)

Supplementary material 10 (DOCX 23 KB)

Supplementary material 11 (DOCX 19 KB)

Supplementary material 12 (DOCX 19 KB)

Supplementary material 13 (XLSX 47 KB)
