# Supplementary material for: Identification and characterization of metabolite quantitative trait loci in tomato leaves and comparison with those reported for fruits and seeds
Source: Metabolomics. 2019 Mar 15;15(4):46. doi: 10.1007/s11306-019-1503-8 (PMC6420416; doi:10.1007/s11306-019-1503-8)
Supplement: Supplementary file 2 — Supplementary material 2 (PPTX 711 KB) [file 11306_2019_1503_MOESM2_ESM.pptx]

## Slide 1
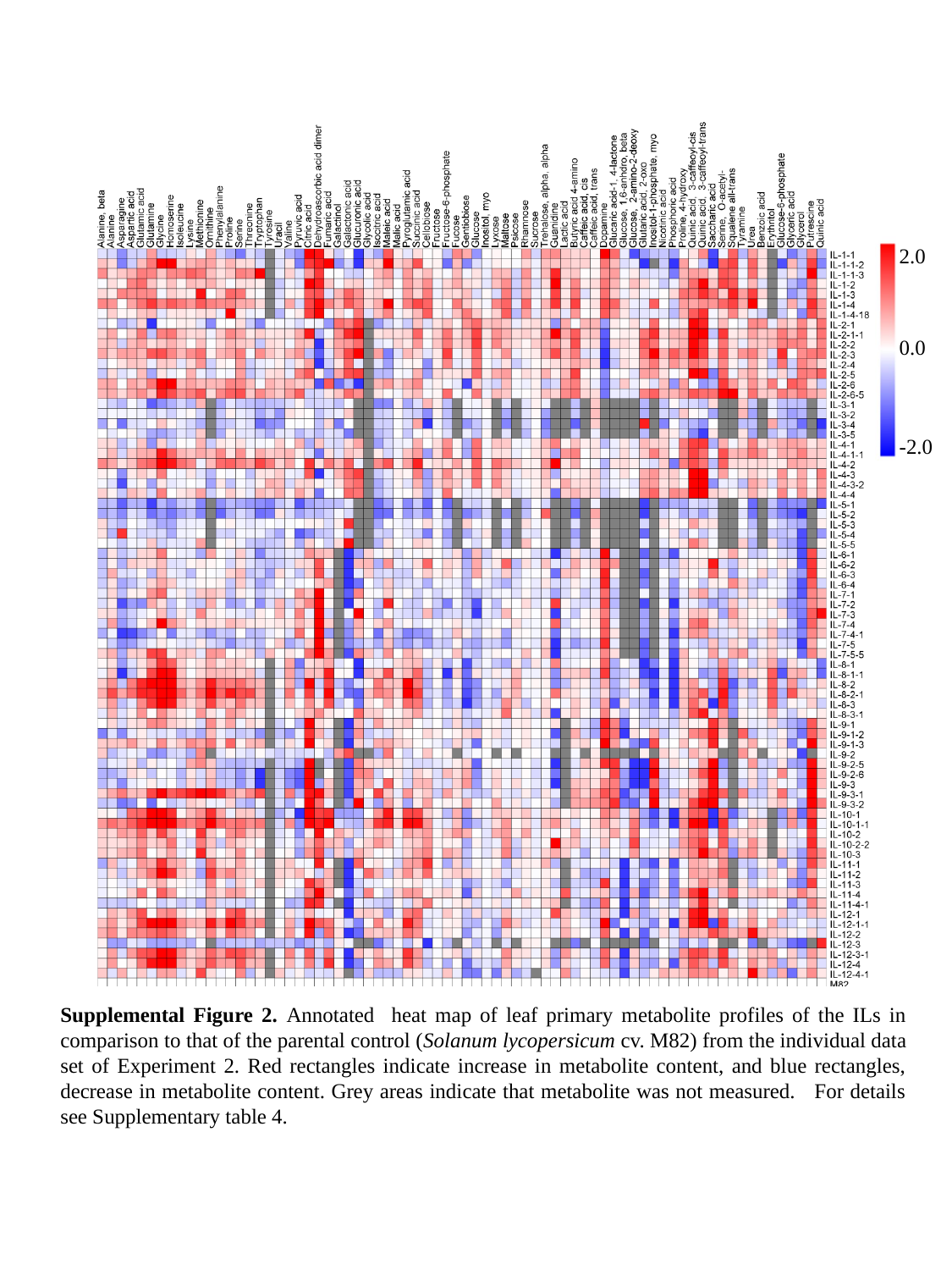

2.0
0.0
-2.0
Supplemental Figure 2. Annotated heat map of leaf primary metabolite profiles of the ILs in comparison to that of the parental control (Solanum lycopersicum cv. M82) from the individual data set of Experiment 2. Red rectangles indicate increase in metabolite content, and blue rectangles, decrease in metabolite content. Grey areas indicate that metabolite was not measured. For details see Supplementary table 4.
