# Supplementary material for: Identification and characterization of metabolite quantitative trait loci in tomato leaves and comparison with those reported for fruits and seeds
Source: Metabolomics. 2019 Mar 15;15(4):46. doi: 10.1007/s11306-019-1503-8 (PMC6420416; doi:10.1007/s11306-019-1503-8)
Supplement: Supplementary file 3 — Supplementary material 3 (PPTX 296 KB) [file 11306_2019_1503_MOESM3_ESM.pptx]

## Slide 1
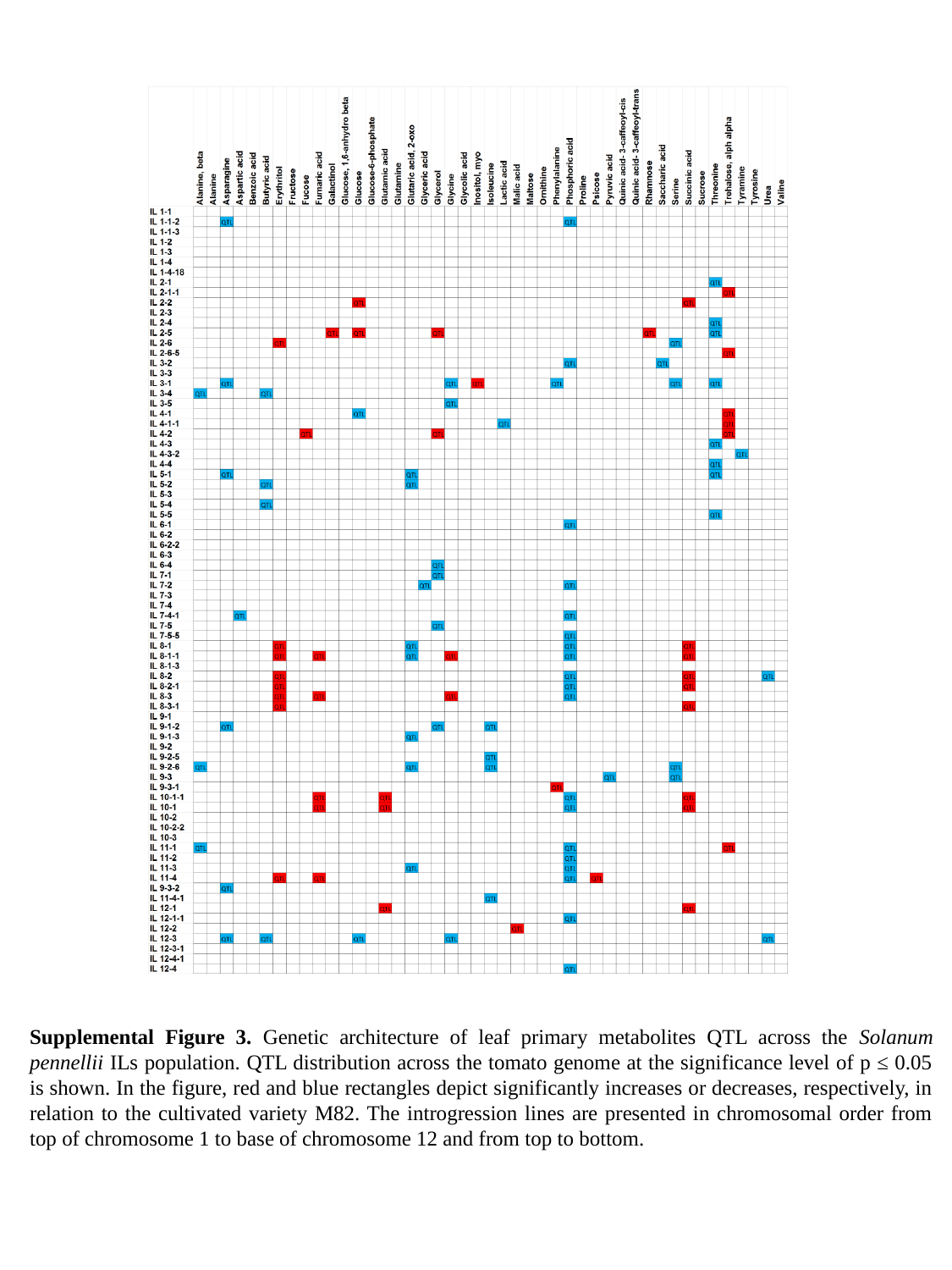

Supplemental Figure 3. Genetic architecture of leaf primary metabolites QTL across the Solanum pennellii ILs population. QTL distribution across the tomato genome at the significance level of p ≤ 0.05 is shown. In the figure, red and blue rectangles depict significantly increases or decreases, respectively, in relation to the cultivated variety M82. The introgression lines are presented in chromosomal order from top of chromosome 1 to base of chromosome 12 and from top to bottom.
