# Supplementary material for: Identification and characterization of metabolite quantitative trait loci in tomato leaves and comparison with those reported for fruits and seeds
Source: Metabolomics. 2019 Mar 15;15(4):46. doi: 10.1007/s11306-019-1503-8 (PMC6420416; doi:10.1007/s11306-019-1503-8)
Supplement: Supplementary file 4 — Supplementary material 4 (PPTX 90 KB) [file 11306_2019_1503_MOESM4_ESM.pptx]

## Slide 1
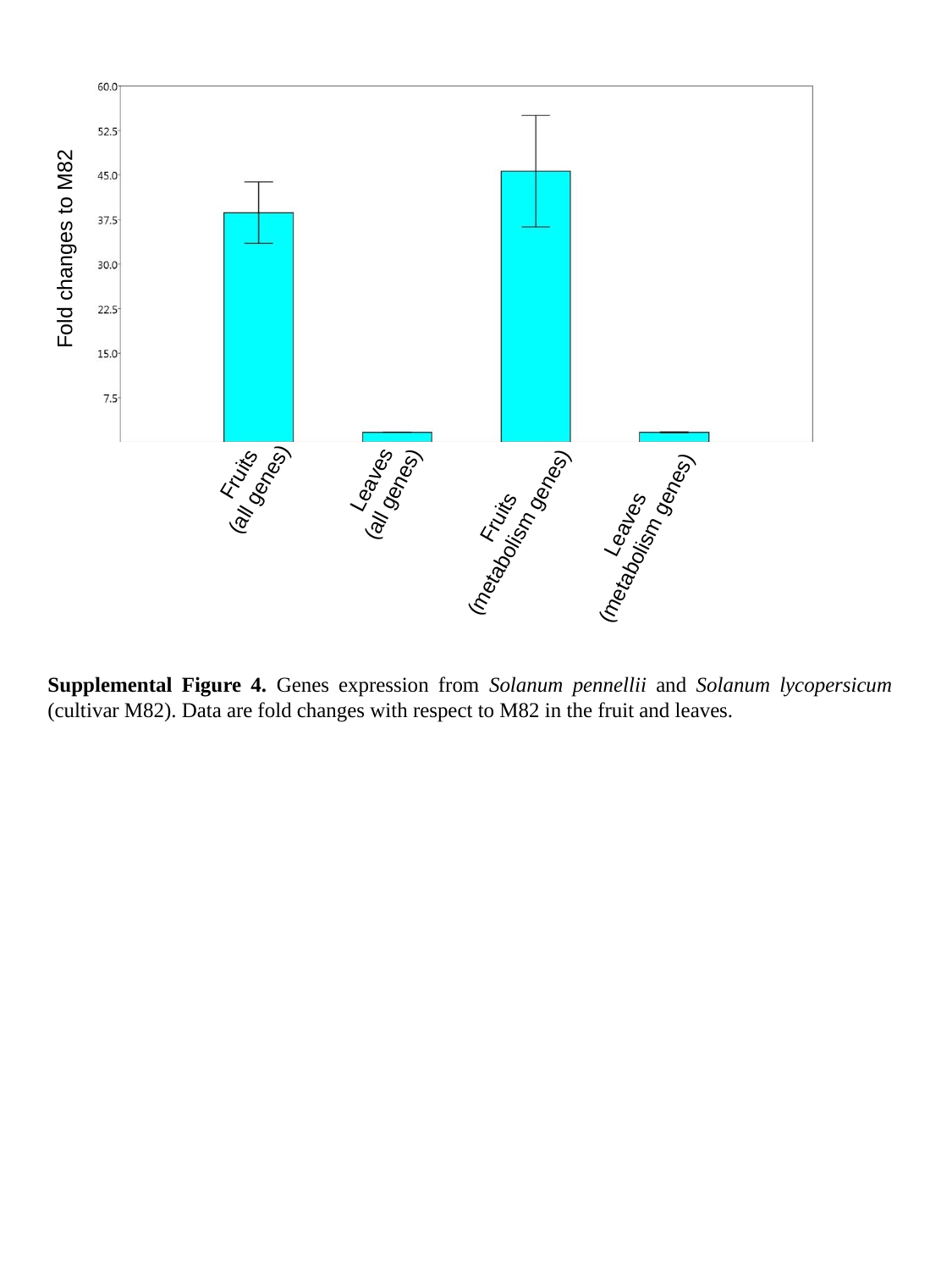

Fold changes to M82
Fruits
(all genes)
Leaves
(all genes)
Fruits
(metabolism genes)
Leaves
(metabolism genes)
Supplemental Figure 4. Genes expression from Solanum pennellii and Solanum lycopersicum (cultivar M82). Data are fold changes with respect to M82 in the fruit and leaves.
