# Supplementary material for: Identification and characterization of metabolite quantitative trait loci in tomato leaves and comparison with those reported for fruits and seeds
Source: Metabolomics. 2019 Mar 15;15(4):46. doi: 10.1007/s11306-019-1503-8 (PMC6420416; doi:10.1007/s11306-019-1503-8)
Supplement: Supplementary file 10 — Supplementary material 10 (DOCX 23 KB) [file 11306_2019_1503_MOESM10_ESM.docx]

**Supplementary table 6.** Number of *Solanum pennellii* introgression lines (ILs) significantly different from the control M82 and percentage of ILs with the same changes in both Experiment 1 and 2 for each metabolite measured in leaf samples harvested from four week old tomato plants.

| **Metabolites** | **Nº of ILs with changes** | | **Nº of ILs with the same changes in Exp1 and 2** | **Total changes** | **% of ILs with conserved changes** |
| --- | --- | --- | --- | --- | --- |
|  | **Exp1** | **Exp2** |  |  |  |
| ***Amino acids*** |  |  |  |  |  |
| β-Alanine | 44 | 5 | 2 | 49 | 4.1 |
| Alanine | 9 | 6 | 0 | 15 | 0.0 |
| Asparagine | 51 | 10 | 6 | 61 | 9.8 |
| Aspartate | 26 | 10 | 2 | 36 | 5.6 |
| Glutamate | 18 | 17 | 3 | 35 | 8.6 |
| Glutamine | 12 | 13 | 1 | 25 | 4.0 |
| Glycine | 38 | 19 | 3 | 57 | 5.3 |
| Phenylalanine | 14 | 10 | 1 | 24 | 4.2 |
| Proline | 32 | 8 | 3 | 40 | 7.5 |
| Serine | 63 | 16 | 4 | 79 | 5.1 |
| Threonine | 54 | 11 | 7 | 65 | 10.8 |
| Tyramine | 37 | 5 | 0 | 42 | 0.0 |
| Valine | 15 | 9 | 0 | 24 | 0.0 |
| GABA | 54 | 5 | 4 | 59 | 6.8 |
| ***Organic acids*** |  |  |  |  |  |
| Fumarate | 5 | 10 | 0 | 15 | 0.0 |
| 2-oxoglutarate | 38 | 15 | 9 | 53 | 17.0 |
| Glycerate | 20 | 13 | 1 | 33 | 3.0 |
| Malate | 36 | 5 | 2 | 41 | 4.9 |
| Pyruvate | 17 | 5 | 0 | 22 | 0.0 |
| Succinate | 33 | 19 | 7 | 52 | 13.5 |
| Saccharate | 68 | 7 | 1 | 75 | 1.3 |
| ***Sugars*** |  |  |  |  |  |
| Fructose | 30 | 1 | 0 | 31 | 0.0 |
| Glucose | 40 | 13 | 5 | 53 | 9.4 |
| Maltose | 10 | 11 | 1 | 21 | 4.8 |
| Sucrose | 17 | 0 | 0 | 17 | 0.0 |
| ***Others*** |  |  |  |  |  |
| α, α´- Trehalose | 35 | 14 | 5 | 49 | 10.2 |
| Myo-inositol | 23 | 4 | 1 | 27 | 3.7 |
| Glycerol | 27 | 17 | 6 | 44 | 13.6 |
| Phosphoric acid | 63 | 24 | 18 | 87 | 20.7 |
| Quinic acid. 3-caffeoyl-. cis- | 19 | 20 | 6 | 39 | 15.4 |
| Quinic acid. 3-caffeoyl-. trans- | 21 | 14 | 1 | 35 | 2.9 |
| Urea | 26 | 2 | 0 | 28 | 0.0 |
