# Supplementary material for: Identification and characterization of metabolite quantitative trait loci in tomato leaves and comparison with those reported for fruits and seeds
Source: Metabolomics. 2019 Mar 15;15(4):46. doi: 10.1007/s11306-019-1503-8 (PMC6420416; doi:10.1007/s11306-019-1503-8)
Supplement: Supplementary file 11 — Supplementary material 11 (DOCX 19 KB) [file 11306_2019_1503_MOESM11_ESM.docx]

**Supplementary table 7.** Coefficient of environment and genotypic variances (C*ve* and C*vg* respectively) of leaf metabolite traits in Experiment 1 (Leiden, Netherlands) and 2 (Buenos Aires, Argentina) of a population of introgression lines of *Solanum pennellii* into a genetic background of *Solanum lycopersicum* (M82).

| ***Metabolite*** | **Experiment 1** | |  | **Experiment 2** | |
| --- | --- | --- | --- | --- | --- |
|  | *Cve* | *Cvg* |  | *Cve* | *Cvg* |
| ***Amino acids*** |  |  |  |  |  |
| β-Alanine | 5.82 | 94.18 |  | 35.41 | 64.59 |
| Alanine | 38.9 | 61.1 |  | 36.54 | 63.46 |
| Asparagine | 12.61 | 87.39 |  | 21.13 | 78.87 |
| Aspartate | 29.2 | 70.8 |  | 35.95 | 64.05 |
| GABA | 15.23 | 84.77 |  | 31.21 | 68.79 |
| Glutamate | 23.89 | 76.11 |  | 30.08 | 69.92 |
| Glutamine | 40.94 | 59.06 |  | 45.7 | 54.3 |
| Glycine | 30.63 | 69.37 |  | 29.66 | 70.34 |
| Phenylalanine | 27.62 | 72.38 |  | 18.69 | 81.31 |
| Proline | 83.39 | 16.61 |  | 45.64 | 54.36 |
| Serine | 9.89 | 90.11 |  | 36.2 | 63.8 |
| Threonine | 17.61 | 82.39 |  | 29.81 | 70.19 |
| Tyramine | 6.8 | 93.2 |  | 33.67 | 66.33 |
| Valine | 27.49 | 72.51 |  | 29.32 | 70.68 |
| ***Sugars*** |  |  |  |  |  |
| Fructose | 30.55 | 69.45 |  | 10.87 | 89.13 |
| Glucose | 25.65 | 74.35 |  | 33.95 | 66.05 |
| Maltose | 27.54 | 72.46 |  | 56.46 | 43.54 |
| Sucrose | 1.6 | 98.4 |  | 7.04 | 92.96 |
| ***Organic acids*** |  |  |  |  |  |
| Pyruvate | 32.56 | 67.44 |  | 15.64 | 84.36 |
| 2-oxoglutarate | 10.06 | 89.94 |  | 7.99 | 92.01 |
| Succinate | 18.23 | 81.77 |  | 23.73 | 76.27 |
| Fumarate | 21.38 | 78.62 |  | 18.54 | 81.46 |
| Malate | 11.76 | 88.24 |  | 10.1 | 89.9 |
| Glycerate | 28.86 | 71.14 |  | 19.15 | 80.85 |
| Saccharate | 11.33 | 88.67 |  | 10.53 | 89.47 |
| ***Others*** |  |  |  |  |  |
| Glycerol | 18.32 | 81.68 |  | 35.54 | 64.46 |
| Myo-inositol | 10.99 | 89.01 |  | 11.54 | 88.46 |
| Phosphoric acid | 8.17 | 91.83 |  | 19.44 | 80.56 |
| Quinic acid 3 caffeoyl cis | 16.44 | 83.56 |  | 48.84 | 51.16 |
| Quinic acid 3 caffeoyl trans | 13.86 | 86.14 |  | 38.74 | 61.26 |
| α, α´- Trehalose | 40.85 | 59.15 |  | 22.11 | 77.89 |
| Urea | 18.94 | 81.06 |  | 7.82 | 92.18 |
