# Supplementary material for: Identification and characterization of metabolite quantitative trait loci in tomato leaves and comparison with those reported for fruits and seeds
Source: Metabolomics. 2019 Mar 15;15(4):46. doi: 10.1007/s11306-019-1503-8 (PMC6420416; doi:10.1007/s11306-019-1503-8)
Supplement: Supplementary file 12 — Supplementary material 12 (DOCX 19 KB) [file 11306_2019_1503_MOESM12_ESM.docx]

**Supplementary table 8.** Predicted gene content within the introgressed regions of lines harboring the identified mQTLs (Figure 3). The gene number was calculated based on Chitwood et al.’s study (2013).

| **Chromosome** | **IL** | **Number of genes** |
| --- | --- | --- |
| **1** | **IL 1-1-2** | 112 |
| **2** | **IL 2-1** | 935 |
|  | **IL 2-1-1** | 357 |
|  | **IL 2-2** | 255 |
|  | **IL 2-4** | 1187 |
|  | **IL 2-5** | 1251 |
|  | **IL 2-6** | 488 |
|  | **IL 2-6-5** | 165 |
| **3** | **IL 3-1** | 191 |
|  | **IL 3-2** | 186 |
|  | **IL 3-4** | 795 |
|  | **IL 3-5** | 485 |
| **4** | **IL 4-1** | 578 |
|  | **IL 4-1-1** | 98 |
|  | **IL 4-2** | 1549 |
|  | **IL 4-3** | 2049 |
|  | **IL 4-3-2** | 1403 |
|  | **IL 4-4** | 472 |
| **5** | **IL 5-1** | 2185 |
|  | **IL 5-2** | 141 |
|  | **IL 5-4** | 169 |
|  | **IL 5-5** | 254 |
| **6** | **IL 6-1** | 1268 |
|  | **IL 6-4** | 150 |
| **7** | **IL 7-1** | 262 |
|  | **IL 7-2** | 1069 |
|  | **IL 7-4-1** | 1515 |
|  | **IL 7-5** | 368 |
|  | **IL 7-5-5** | 335 |
| **8** | **IL 8-1** | 1337 |
|  | **IL 8-1-1** | 1256 |
|  | **IL 8-2** | 910 |
|  | **IL 8-2-1** | 695 |
|  | **IL 8-3** | 389 |
|  | **IL 8-3-1** | 128 |
| **9** | **IL 9-1-2** | 258 |
|  | **IL 9-1-3** | 545 |
|  | **IL 9-2-6** | 360 |
|  | **IL 9-3** | 721 |
|  | **IL 9-3-1** | 509 |
|  | **IL 9-3-2** | 315 |
| **10** | **IL 10-1** | 1920 |
|  | **IL 10-1-1** | 319 |
| **11** | **IL 11-1** | 593 |
|  | **IL 11-2** | 1703 |
|  | **IL 11-3** | 1520 |
|  | **IL 11-4** | 131 |
|  | **IL 11-4-1** | 103 |
| **12** | **IL 12-1** | 226 |
|  | **IL 12-1-1** | 111 |
|  | **IL 12-2** | 1595 |
|  | **IL 12-3** | 2069 |
|  | **IL 12-4** | 458 |
